# Examining systemic steroid Use in older inflammatory bowel disease patients using hurdle models: a cohort study

**DOI:** 10.1186/s40360-015-0034-9

**Published:** 2015-12-08

**Authors:** Sophia L. Johnson, Mari Palta, Christie M. Bartels, Carolyn T. Thorpe, Jennifer M. Weiss, Maureen A. Smith

**Affiliations:** Pharmaceutical Health Services Research Department, University of Maryland School of Pharmacy, 220 Arch Street, 12th Floor, Room 01-218, Baltimore, MD 21201 USA; Department of Population Health Sciences, University of Wisconsin School of Medicine and Public Health, 610 Walnut Street, 53726 608-263-2520, Madison, Wisconsin USA; Department of Biostatistics & Medical Informatics, University of Wisconsin School of Medicine and Public Health, Madison, Wisconsin USA; Department of Medicine, Rheumatology Division, University of Wisconsin School of Medicine and Public Health, 800 Highland Avenue, 608-263-3457, Madison, Wisconsin USA; Center for Health Equity Research and Promotion, Veterans Affairs Pittsburgh Medical Center, 3501 Terrace Street, 412-624-7794, Pittsburgh, Pennsylvania USA; Department of Pharmacy and Therapeutics, University of Pittsburgh School of Pharmacy, Pittsburgh, Pennsylvania USA; Department of Medicine, Division of Gastroenterology and Hepatology, University of Wisconsin School of Medicine and Public Health, 1685 Highland Avenue, Room 4230, 608-263-1995, Madison, Wisconsin USA; Department of Family Medicine, University of Wisconsin School of Medicine and Public Health, Madison, Wisconsin USA; Department of Surgery, University of Wisconsin School of Medicine and Public Health, Madison, Wisconsin USA

**Keywords:** Negative binomial-logit hurdle models, Zero-altered two part models, Steroids, Inflammatory Bowel Disease, Older Patients

## Abstract

**Background:**

Interpreting clinical guideline adherence and the appropriateness of medication regimens requires consideration of individual patient and caregiver factors. Factors leading to initiation of a medication may differ from those determining continued use. We believe this is the case for systemic steroid therapy in inflammatory bowel disease (IBD), resulting in a need to apply methods that separately consider factors associated with initiation and duration of therapy. To evaluate the relationship between patient characteristics and the frequency and duration of incident steroid use we apply a 2-part hurdle model to Medicare data. We do so in older patients with tumor necrosis factor antagonist (anti-TNFs) contraindications, as they are of special interest for compliance with Medicare-adopted, quality metrics calling for anti-TNFs and nonbiologic immune therapies to reduce steroid utilization. Many older patients have contraindications to anti-TNFs. However, nonbiologics cause adverse events that are concerning in older adults, limiting their use in this population and increasing reliance on systemic steroids.

**Methods:**

We used a national Medicare sample for 2006–2009 including patients with 12 months or greater of Parts A and B and 6 months or greater of Part D coverage, IBD confirmed with at least 2 claims for ICD-9CM 555.xx or 556.xx, anti-TNF contraindications and without contraindications to nonbiologic agents. We applied a negative binomial-logit hurdle model to examine patient characteristics associated with systemic steroid utilization.

**Results:**

Among the 1,216 IBD patients without baseline steroid use, 21 % used systemic steroids. Odds of receiving systemic steroids were greater in those younger, rural, and those receiving other agents. Available patient characteristics failed to predict longer steroid treatment duration.

**Conclusions:**

Our study identified differences in predictors of frequency and duration of medication use and suggests the utility of two-part models to examine drug utilization patterns. Applying such a model to Medicare data, we determined that despite medical consensus that systemic steroid use should be minimized, its use was substantial. Findings indicate anticipated difficulties in implementing recently adopted quality measures to avoid systemic steroids.

## Background

Beyond occasional ulcerative colitis patients that can be managed with aminosalicylates, patients with moderate to severe inflammatory bowel disease (IBD) require the use of systemic steroids or “steroid-sparing” regimens including tumor necrosis factor antagonists (anti-TNFs) or nonbiologic immunomodulators (nonbiologics [e.g. thiopurines, methotrexate]) to control their symptoms. [1–3] However, systemic steroids do not maintain disease remission, relegating their appropriateness to the induction of remission [4, 5]. Furthermore, many steroid-associated adverse events are duration dependent. The implication of steroid initiation and of their continued use therefore differ, as may the patient and caregiver characteristics determining short and long term steroid management. Identifying these characteristics is important, though direct drug costs of systemic steroids are minimal, the health burden is considerable. Approximately 10 % of all reported drug adverse events in the US are associated with systemic steroids according to Healthcare Cost and Utilization Project (HCUP) analyses [6]. Such complications are particularly costly in older patients who are at greater baseline risk [6–8].

Steroid-sparing regimens are associated with deep, sustained remission and improved health outcomes as supported by clinical, endoscopic and biomarker evidence [1, 9, 10]. However, they may be expensive and these medications also have adverse events such as infections and malignancies [1, 9–14]. Considering the risks and benefits associated with IBD drugs [15], the Center for Medicare and Medicaid Services (CMS), the administrator for Medicare, a universal health insurance program for US citizens ≥65, adopted IBD-specific quality measures that call for the use of steroid-sparing maintenance regimens [16]. Specifically, steroid use for ≥ 60 days or disease that requires multiple steroid courses should result in the initiation of steroid-sparing agents [5, 15, 16]. However, the limited data available suggest low utilization rates for anti-TNFs and nonbiologic immunomodulators compared to high steroid use (9.5 % vs 31 %) in older patients [17].

The low frequency of anti-TNF utilization, and, thereby, greater use of systemic steroids, may be partly attributable to higher rates of anti-TNF contraindications (decompensated congestive heart failure (CHF), malignancies) in older patients compared to their younger counterparts [18, 19]. For such patients, nonbiologics remain a guideline-recommended steroid-sparing option. Still systemic steroids may be preferred over nonbiologics by patients and providers. Understanding drug selection patterns for patients with anti-TNF contraindications is critical in light of the new quality measure, but no information has been published on this population.

In this paper we examine IBD drug utilization, and patient characteristics associated with steroid initiation and the duration of steroid use in a nationally representative sample of older patients with anti-TNF contraindications, but without nonbiologic contraindications (hematologic malignancies, liver disease). We conducted the steroid analysis using an approach that is underutilized in the medical literature, the negative binomial logit hurdle model. We performed this evaluation using data from the period between the release of the medical position statement [15] supporting the new measure and its adoption by CMS [16] so that our findings may serve as a baseline snapshot for future examination of the impact of this national policy change. While logistic regression has been used to identify patient characteristics associated with the frequency of systemic steroid utilization in multiple sclerosis patients [20], to our knowledge a two-part model has not previously been used to examine the association between patient characteristics and the frequency and duration of incident steroid use. However, using less frequently deployed analytic methods to examine medication use is important to determining the value of these models for such purposes.

## Methods

### Study Sample

We examined claims and enrollment data for Medicare fee-for-service (FFS) beneficiaries, which represent approximately 88 % of all Medicare recipients, to identify a sample of older adults with a diagnosis of IBD and contraindications to anti-TNF therapy [21]. Patients ≥65 years old with at least 12 months of Parts A and B (hospital and medical visit) and 6 months of Part D (outpatient prescription) coverage during the years 2006–2009 were included. Data were included for up to 6 months prior to coverage by Medicare D. IBD diagnosis was ascertained using a case-finding algorithm (≥2 claims for appropriate International Classification of Diseases, 9^th^ edition (ICD-9) codes [Crohn’s Disease: 555.xx] or [Ulcerative Colitis: 556.xx]) [22, 23]. The first 12 months of data are referred to as collected during the “baseline year” and began after the patient had at least one IBD claim. Data collected after the baseline period was considered from the “follow-up period”. Follow-up continued until December 31, 2009, disenrollment from Medicare Parts A, B or D or death, whichever occurred first. Patients were excluded if they did not have a confirmatory IBD claim by the end of the follow-up period. Drug contraindications were determined during the baseline year. Contraindication to anti-TNF therapy was defined as advanced CHF or malignancy. Advanced CHF was identified as ≥1 outpatient claim for a CHF diagnosis (ICD-9 398.91, 402.01, 402.11, 402.91, 404.01, 404.03, 404.11, 404.13, 404.91, 404.93, 414.8, 428.x) and at least 1 CHF hospitalization (primary inpatient discharge diagnosis code for CHF [ICD-9 codes: 398.91, 404.x1, 404.x3, 428.0–428.9]) [24]. Both solid tumor and hematologic malignancies were ascertained using the 2008 Elixhauser criteria, version 3.3 (ICD-9 codes: 140.0–172.9, 174.0–175.9, 179–195.8, 258.01–258.03, 196.0–199.1, 789.51, 200.00–202.38, 202.50–203.01, 203.8–203.81, 238.6, 273.3) [25]. Patients with nonbiologic contraindications comprising hematologic malignancies (defined above) and liver disease (ICD-9 codes for 571.0–571.9, 070.2–070.9, 572.2–572.4) [26] were excluded from study as they were not eligible to receive any steroid- sparing agent.

### Outcome Drug Class Variables

The primary outcome variables were receipt and duration of systemic steroid therapy (prednisone, methylprednisolone, budesonide). Although the entire class of agents were included as an outcome variable, prednisone represented >95 % of all incident steroid use in this study. Systemic steroid use was identified from Medicare Part D [27] claims history during the time the patient had Part D coverage, by National Drug Codes (NDCs) using information on NDCs and therapeutic class in the Multum Lexicon™ Plus database (Cerner Multum Incorporated, Denver, Colorado). A patient day dataset was constructed with patients assigned to having received therapy on a given day based upon the ReComp algorithm [28]. Identifying drug administration days allowed computing period prevalence, treatment duration and incident drug use.

Incident steroid use was defined as a new claim for systemic steroids that started during the follow-up period, and steroid therapy days included all the days systemic steroids were received (regardless of gaps in treatment) during the follow-up. Prevalent systemic steroid use is included in the descriptive analysis only.

### Explanatory Drug Class Variables

The use of home administered anti-TNF infusions (infliximab), anti-TNF injections (adalimumab), non-biologic immunomodulators, aminosalicylates, locally administered steroids and antidiarrheal therapies were also identified from Medicare Part D [27] claims history during the baseline period by the approach used for the systemic steroid outcome variable. Facility-administered anti-TNF infusions (infliximab) were ascertained from Part A and B claims for Healthcare Common Procedure Coding System (HCPCS) J-code 1745 [27, 29]. These therapies were assessed during the baseline period and included as predictors in the model for steroid use.

### Other Explanatory Variables

Demographic information was obtained from the Medicare denominator file. This file was used to determine Medicaid coverage status. The sample of Medicare beneficiaries was merged with Census 2010 Summary File 3 (SF3) data (yielding socioeconomic characteristics on households). Patient zip-codes were used to assign urban status based on rural urban commuting area (RUCA) codes [30].

Patient medical and health care characteristics were ascertained from the Medicare data during the baseline period, and included a comorbidity index (Charlson index), IBD disease severity (endoscopies, surgeries), and health resource utilization (managing provider type, hospitalization, emergency department visit).

The primary provider type (primary care provider, gastroenterologist or other specialists) for IBD management was assigned as the provider with the greatest number of evaluation and management (E&M) IBD visits (Appendix 1) [31]. Assuming patients may receive one surveillance endoscopy annually [32, 33], we considered >1 endoscopy (identified on outpatient and inpatient claims as ICD-9 codes and on carrier claims as CPT codes, Appendix 1) [22] an indicator of disease severity. IBD surgeries were identified from inpatient claims for an appropriate ICD-9 procedure code (Appendix 1) [34]. Other health resource use (hospitalizations, emergency department visits), and comorbidity indices were determined from ICD-9 codes and HCPCS codes from inpatient claims, carrier claims and E&M visits, as appropriate [35].

### Statistical Analysis

We provide descriptive information on all drug classes considered and model the use of incident steroid use. Patients who received systemic steroid therapy during the baseline period were excluded from regression analyses.

Since the deployment of any systemic steroids is important and the duration of steroid therapy is separately an important indicator of appropriate use, we employ a hurdle model in the analysis of incident steroid use [36]. The logistic portion of the model evaluated patient factors associated with being an incident steroid recipient. Furthermore, it is likely that individuals vary in their propensity to continue on systemic steroids due to unmeasured factors, thereby generating overdispersion and making the negative binomial a more appropriate choice for length of steroid treatment than the Poisson. The negative binomial portion was truncated at zero and assessed factors associated with steroid therapy days among incident steroid users.

**Fig. 1:**
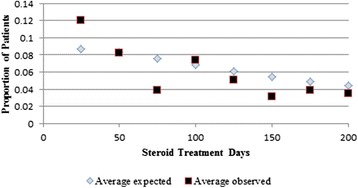
Data Fit for Count Model. The average total number of days of steroid therapy (observed) among patients who received steroid therapy and the average total number of steroid therapy days that steroid recipients were expected to receive drug therapy based on their characteristics

Robust standard errors were used for statistical inference on regression coefficients. Because of the relatively small sample size, forward step-wise model building was employed for the hurdle model components with sociodemographic characteristics and IBD drug classes in the first 12 months included in the initial model and each candidate covariate of reasonable cell size (>10) considered. Candidate covariates with descriptive importance (eg. region) and/or marginal statistical significance (*p* < 0.1) were retained in the final models. Model fit for the logistic part of the model was evaluated by comparing deciles of observed and predicted percentage receiving systemic steroids using the Hosmer-Lemeshow goodness of fit statistics (*p* = ns). Fit of the expected number of observations from a negative binomial to the data is presented graphically (Figure 1, Chi square *p* = ns).

This study included a dynamic cohort with varying patient follow-up times allowing some participant’s greater time to receive systemic steroids and additional steroid therapy days; therefore, time-offsets were used in all models. The logistic time offset was defined as the natural log of the time from the beginning of the entire observation period until systemic steroids were initiated for steroid recipients or until the end of follow-up for non-recipients. For the count model, the time offset was the time from the beginning of the entire observation period until the end of follow-up for a given patient. Our time off-set mirrors the hurdle model weighting approach used by Senturk and colleagues in their examination of cardiovascular events in the dialysis population [37].

The results of the hurdle model were compared to those of fitting a Poisson model with the log link and robust standard errors to the total number of days on systemic steroids, which is the model frequently used in medical literature [38]. The same covariates were included in the Poisson and hurdle models and significance levels compared. We evaluated the Poisson model fit using the Schwartz Bayesian information criterion and the degree of over-dispersion using deviance/df.

Since the complementary log-log (CLL) link better corresponds to a binary analysis in which time is a consideration in the probability of event occurrence, a sensitivity analysis of the logistic component of the hurdle model was performed using the CLL link for the generalized linear regression of incident steroid utilization, and generated similar findings (not reported).

Statistical analysis was conducted using Stata version 13 (StataCorp, College Station, TX) and results are presented as odds ratios (OR), ratio of durations (count model), and 95 % Confidence Intervals (CI).

This retrospective cohort study was determined to be exempt from oversight by the University of Wisconsin Institutional Review Board.

## Results

### Descriptive Characteristics of Full Study Sample

Eighteen percent (*n* = 1,860) of the full cohort of Medicare FFS beneficiaries aged 65+ with IBD (*n* = 10,362) had anti-TNF contraindications. Of these, 9.6 % (*n* = 178) also had nonbiologic contraindications, resulting in a final study sample of 1,682. Overall, participants had a mean age of 79 years (*sd* = 7.7), 68 % were female, 88 % white and 76 % resided in an urban or suburban area (Table 1). Aminosalicylates, locally administered steroids and antidiarrheals were used by 22 %, 1 % and 12 % of study participants at baseline, respectively. Forty-three percent of participants (*n* = 726) received systemic steroid therapy after starting the study. There were 466 baseline users and 260 incident users who were the focus of the regression analysis whereas only 5 % (*n* = 85) received nonbiologic immunomodulators, and 2 % received anti-TNF therapy despite having anti-TNF contraindications.

**Table 1:** Baseline Participant Characteristics, Overall and by Drug Therapy

	Full cohort (*n* = 1682)		Ever systemic steroid users (*n* = 726)^a^		Ever nonbiologic users (*n* = 85)^a^	
Sociodemographics^b^	n	%	n	%	n	%
Age Mean (SD)^c^		79 (8)		78 (7)^e^		75 (7)^e^
Female	1136	68	494	68	50	59
Caucasian	1474	88	654	90^d^	80	94
Region						
Northeast	444	26	182	25	19	22
Southeast	459	27	208	29	24	28
Midwest	417	25	189	26	28	33
Rocky Mountain	34	2	12	2	<11^g^	<11^g^
Southwest	163	10	96	10	<11^g^	<11^g^
Pacific (incl HI, AK, PR)^h^	165	10	68	9	<11^g^	<11^g^
Urban/Suburban	1266	76	516	72^e^	61	72
Medicaid coverage	547	33	219	30	21	25
Clinical^b^	n	%	n	%	n	%
Polypharmacy (>5 drugs)	1290	81	602	85^e^	69	83
Charlson Index Mean (SD)		4 (3)		4 (3)		4 (2)
Primary managing provider type						
Primary Care Provider	769	46	320	44.4	38	44.7
Gastroenterologist	19	1	<11^g^	<11^g^	<11^g^	<11^g^
Other specialists	868	52	393	55	44	52
antiTNF	37	2	26	4^e^	18	21^e^
Aminosalicylates	374	22	215	30^e^	38	45^e^
Locally administered steroids	12	1	<11^g^	<11^d,g^	<11^g^	<11^g^
Antidiarrheals	209	12	111	15^e^	14	17
>1 endoscopy	452	27	201	28	25	29
IBD surgery	106	6	41	6	<11^g^	<11^g^
Hospitalizations Mean (SD)		2 (2)		2 (2)^e^		2 (2)
ED visits Mean (SD)		1 (2)		1 (2)^e^		1 (2)

^a^Bivariate analyses is the comparison of Steroid Use to NonUse; Nonbiologic Use to Nonuse; ^b^Baseline characteristics were ascertained during 12 months prior to study inclusion; ^c^SD = standard deviation ^d^ *p* < .05, ^e^ *p* < .01, ^f^ *p* < .001; ^g^Cell sizes are too small to include variable in regression model & requires cell suppression

^h^HI = Hawaii, AK = Alaska, PR = Puerto Rico

**Fig. 2:**
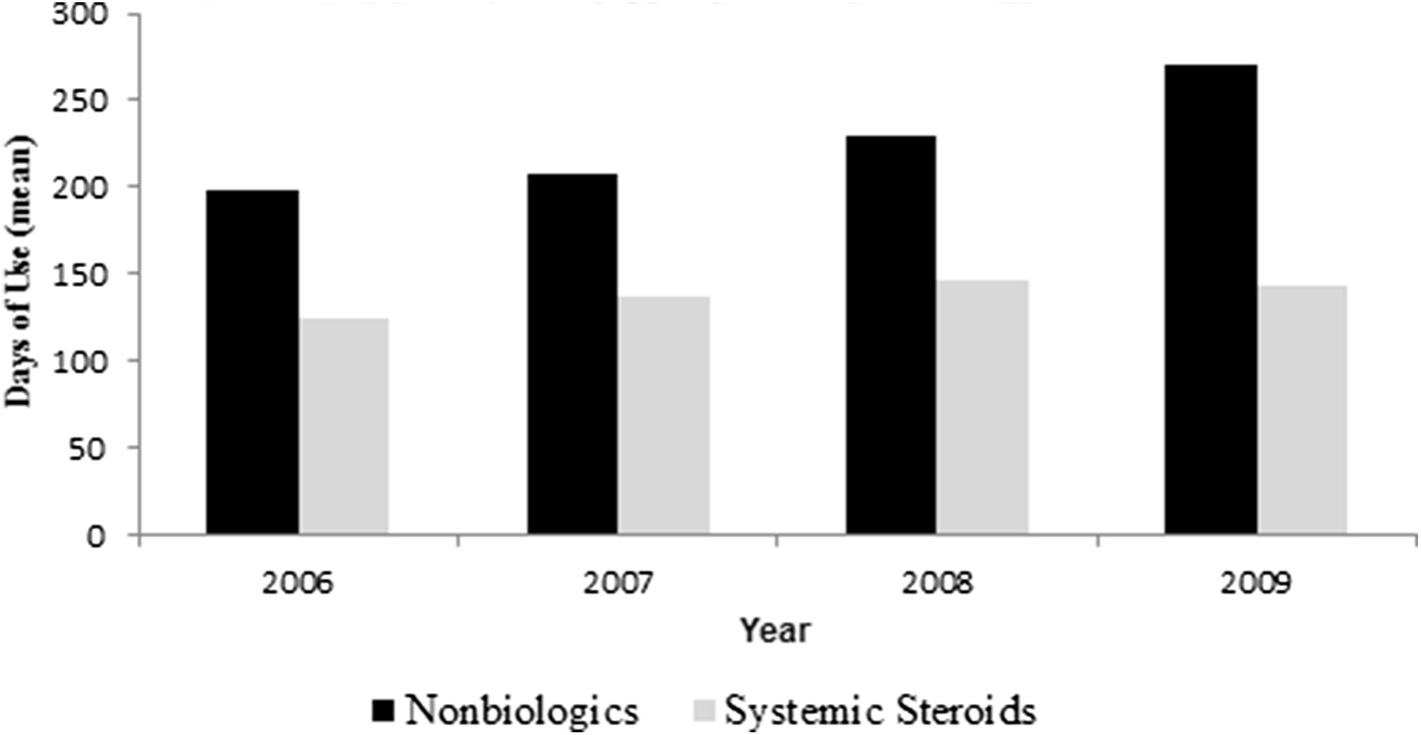
Mean Annual Number of Days on Therapy Among Utilizers. For each year of the study, the number of days that all nonbiologic and systemic steroid therapy recipients received the respective drug therapy

Systemic steroids were the most frequently used class of agents during every year of observation (Table 2). The number of patients treated with nonbiologic immunomodulators was substantially smaller than that of steroid recipients 35–45 users per 1000 patients per year versus 303–345 per 1000 patients per year, respectively. There were more patients taking systemic steroids than nonbiologics, but the average number of days on therapy was shorter for steroid recipients (124–147 days per year) than for patients receiving nonbiologics (199–271 days per year). This is expected given induction steroid therapy practices that call for shorter treatment courses (Figure 2).

**Table 2:** Annual Utilization by IBD Drug Class

	Number of utilizers per 1000 IBD patients per year^a^			
Class of Agent	2006	2007	2008	2009
Systemic Steroids	303	345	336	317
Immunomodulators	48	53	50	45
antiTNFs	12	16	22	17
Nonbiologic Immunomodulators^b^	42	45	37	35
Aminosalicylates	285	265	246	249
Locally administered steroids	12	11	<11^c^	<11^c^
Antidiarrheals	129	121	115	118

^a^Mid-year population used to calculate utilizers per year

^b^Nonbiologic immunomodulators include azathioprine, mercaptopurine, and methotrexate

^c^Cell size too small and requires suppression

^d^These utilization numbers were derived by dividing the number of patients who received a particular drug class during the year by the mid-year study population and multiplying by 1000.

### Multivariable Analyses of Predictors of Incident Steroid Exposure by 2-part Hurdle Model

A total of 1,216 IBD patients who had no steroid use during the baseline period were included in the follow-up analysis. Overall 21 % (*n* = 260) of steroid recipients in the regression analysis cohort were incident users. Patients had greater odds of receiving incident systemic steroids if they were younger (OR = 1.25 per 5 year age decrease, CI = 1.14, 1.39) or lived in rural areas (OR = 1.54, CI = 1.10, 2.13) (Table 3). Steroid use was positively and significantly associated with receipt of aminosalicylates and antidiarrheal therapies in the first 12 months.

**Table 3:** Multivariable Regression Model of Steroid Exposure

	Steroid use (yes/no)^a^		Duration of steroid use among utilizers^b^	
	OR	CI	Ratio of Durations^c^	CI
Age ( 5 year decrease)	1.25	(1.14, 1.39)^f^	0.97	(0.88, 1.06)
Female	1.30	(0.93, 1.80)	1.11	(0.85, 1.45)
Caucasian	1.46	(0.87, 2.46)	0.87	(0.54, 1.38)
Region				
Midwest	1.00	Reference	1.00	Reference
Northeast	0.92	(0.61, 1.37)	1.13	(0.81, 1.57)
Southeast	0.91	(0.61, 1.35)	0.93	(0.67, 1.29)
Southwest	0.80	(0.47, 1.38)	0.84	(0.54, 1.32)
Rocky Mountain	0.44	(0.12, 1.55)	0.90	(0.29, 2.73)
Pacific & HI, AK, PR^g^	0.70	(0.38, 1.30)	0.84	(0.51, 1.40)
Rural	1.54	(1.10, 2.13)^d^	0.93	(0.71, 1.20)
Medicaid eligible	0.80	(0.57, 1.13)	0.89	(0.66, 1.20)
antiTNFs	1.66	(0.52, 5.34)	0.63	(0.30, 1.29)
Nonbiologic immunomodulators	2.14	(0.82, 5.57)	0.76	(0.42, 1.36)
Aminosalicylates	1.78	(1.26, 2.51)^e^	1.01	(0.77, 1.33)
Locally administered steroids	3.77	(0.69, 20.6)	1.41	(0.46, 4.35)
Antidiarrheals	1.72	(1.12, 2.62)^d^	0.83	(0.59, 1.18)
Charlson index	0.96	(0.90, 1.01)	1.00	(0.95, 1.06)
Hospitalizations	0.97	(0.88, 1.07)	0.99	(0.90, 1.09)
Endoscopy (>1)	1.09	(0.76, 1.56)	0.83	(0.60, 1.15)
IBD-associated Surgery	0.75	(0.40, 1.39)	0.89	(0.48, 1.64)

^a^Dependent variable was incident steroid use during the follow-up period, Individuals with baseline steroid use were excluded from this model (*n* = 1216)

^b^Dependent variable was days of steroid use among steroid users (*n* = 260) during the follow-up period, Individuals with baseline steroid use were excluded from this model; count truncated at zero; ^c^Effect measure is ratio of steroid therapy durations; All explanatory variables are measured within the first 12 months after study entry; ^d^ *p* < .05, ^e^ *p* < .01, ^f^ *p* < .001; ^g^HI = Hawaii, AK = Alaska, PR = Puerto Rico

Longer steroid treatment courses were not associated with any of the observed patient characteristics (Table 3).

### Multivariable Analyses of Predictors of Incident Steroid Exposure by Simple Model

When predictors of steroid therapy duration are examined using the simple Poisson regression (Table 4), it is shown that those who are younger receive additional steroid days at a greater rate (RR = 1.19 per 5 year lower age, CI = 1.08, 1.31. Poisson regression demonstrated greater rates of additional days of steroid use among locally administered steroid recipients (RR = 4.39, CI = 1.51, 12.70). The Poisson model, similar to the hurdle model, would have detected the statistically significant inverse relationship between age and steroid use (Table 4) but would have failed to identify rurality and the baseline use of aminosalicylates or antidiarrheals as predictors of steroid use.

**Table 4:** Simple Poisson Multivariable Regression Model of Steroid Exposure

	Poisson regression	
	Duration of Steroid Use	
	RR	CI
Age ( 5 year decrease)	1.19	(1.08, 1.31)^e^
Female	1.40	(0.97, 2.02)
Caucasian	1.39	(0.80, 2.42)
Region		
Midwest	1.00	Reference
Northeast	0.86	(0.56, 1.32)
Southeast	0.71	(0.46, 1.08)
Southwest	0.60	(0.30, 1.18)
Rocky Mountain	0.42	(0.09, 2.05)
Pacific & HI, AK, Pr^f^	0.60	(0.29, 1.23)
Rural	1.23	(0.86, 1.77)
Medicaid eligible	1.04	(0.72, 1.51)
antiTNFs	0.62	(0.19, 2.03)
Nonbiologic immunomodulators	1.58	(0.75, 3.33)
Aminosalicylates	1.41	(0.98, 2.03)
Locally administered steroids	4.39	(1.51, 12.70)^d^
Antidiarrheals	1.07	(0.69, 1.65)
Charlson index	0.96	(0.90, 1.01)
Hospitalizations	0.96	(0.87, 1.05)
Endoscopy (>1)	0.87	(0.60, 1.27)
IBD-associated Surgery	0.68	(0.35, 1.33)

^a^Dependent variable was days of steroid use among participants (*n* = 1216) during the follow-up period; The models were run with robust standard errors, Individuals with baseline steroid use were excluded from these models; ^b^RR = rate ratios; All explanatory variables are measured within the first 12 months after study entry; ^c^ *p* < .05, ^d^ *p* < .01, ^e^ *p* < .001; ^f^HI = Hawaii, AK = Alaska, PR = Puerto Rico

## Discussion

Almost half of the patients in our study received systemic steroids which is greater than expected given that guidelines advocate systemic steroids be reserved for induction therapy and caution against numerous steroid-associated adverse events.

The American Gastroenterological Association (AGA) position underlying the CMS, steroid-sparing quality metric, states that patients receiving ≥ 60 consecutive days of systemic steroids should be initiated on an anti-TNF or nonbiologic unless they have contraindications to those agents [16]. The patients in our study had contraindications to anti-TNF therapy; but were eligible for treatment with nonbiologics. Our findings demonstrate average annual systemic steroid therapy days that are at least double this recommendation, suggesting significant gaps and conflict with the treatment guidelines [16]. The prolonged steroid treatment courses suggest that steroids are frequently used beyond induction. Specifically, these patients are at greater risk for the steroid-associated adverse events seen with longer treatment courses, including hypothalamus pituitary adrenal axis suppression, osteoporotic fractures, coronary artery disease, lipodystrophy, cataracts and potentially serious infections [4, 6]. We found greater initiation of systemic steroids in rural than in urban patients, reflecting a potential disparity.

The use of nonbiologics instead of systemic steroids is an innovation, and innovations are more likely to diffuse and be adopted in cosmopolitan settings with greater interpersonal communication between providers and near peer IBD experts [39]. Additionally, patients who reside in larger, more urban communities may have greater access to support groups and information motivating them to request or agree to nonbiologic immunomodulators [39].

Two-part models such as hurdle models are rarely reported in the medical literature but common in economics. The lack of their use may hamper a comprehensive understanding of the utilization and predictors associated with specific drug therapy regimens. The hurdle model fits the subject matter of steroid use especially well. Importantly receiving any systemic steroids is typically related to the need to abate a disease flare after patients have failed less aggressive drugs (aminosalicylates, locally administered steroids and antidiarrheal therapies) or in settings where newer therapies are not in common use (rural settings). Prolonged use may be related to factors that are more difficult to measure like clinical inertia, patient and clinician preferences, and suboptimal maintenance regimens.

In the current study, we indeed found that these models were not overlapping. This is notable because while none of the studied characteristics were predictive of prolonged use, several factors like being younger and failing weaker drugs predicted steroid initiation which underscores the fact that these two relationships should be modelled separately. The difference was not due to lack of power in the duration model, as confidence intervals were of similar width, and several of the non-significant rate ratios were in the opposite direction from the odds ratios for steroid initiation. Fitting the simple count model would have incorrectly concluded that there is no difference between steroid use in rural versus urban dwellers or for those with several classes of baseline drug use.

It should be noted that a simple Poisson model does not separate predictors of initiation and duration of steroids, as non-use as an outcome is on a continuum of shorter use. While older age was a predictor of steroid initiation, it was not related to duration of use. The Poisson model, while finding age a significant predictor of duration from zero days onwards, does not make that distinction.

Grootendorst showed the utility of a two-part hurdle model for a similar application to examine the effect on prescription drug utilization of removing prescription copays for individuals’ ≥ 65 years old [40]. In that examination, Grootendorst compared the 2-part model to simple models. He found the former to be superior based upon accuracy and model selection criteria. Grootendorst’s work is relevant to the current study because we also sought to evaluate drug utilization and also identified differences between modeling the simple and 2-part models.

The major strengths of our study were our analytic approach and the use of a large national sample allowing us to consider a sub-population of older IBD patients with anti-TNF contraindications to identify drug treatment patterns. Reporting on the use of a 2-part model to examine medication use patterns encourage debate regarding the value of these methods to study complicated utilization patterns. Since we used CMS’ random sample of Medicare beneficiaries we were able to include 1,682 older patients with IBD and anti-TNF contraindications, which allowed us to examine drug utilization in an un-studied age group to establish baseline data to evaluate national policy change. The major limitation to our study was the inability to link steroid use to an indication due to our use of claims data instead of electronic health records. Additionally, we cannot tell if nonbiologics were unsuccessfully tried prior to our investigation and contributed to low use during our study. However, even if patients tried nonbiologics in the past, new data about optimizing nonbiologic regimens may mean these patients were candidates for renewed treatment with nonbiologics during our study period [41, 42].

## Conclusion

Our results indicate the importance of separately considering drug initiation and length of treatment in identifying determinants of use. Systemic steroids were overutilized, and our model identifies rural residence and the baseline use of drug therapies to abate symptoms as risk factors for steroid initiation. However, the use of steroids for long treatment courses represents a quality gap and, potentially, a cost concern given the expense of steroid-associated adverse events and poorly controlled IBD. Our model showed that factors associated with initiation did not predict prolonged steroid use, and identifies this as an area requiring a dataset with more patient specific variables. Two part hurdle models are underutilized for examining duration of therapy in the medical literature but proved to be useful in our consideration of steroid therapy predictors. These models may have wider application to other medication utilization studies in the future.

## Appendix 1

**Table 5:** Variable Definitions

Variable	Identification Codes
IBD encounters to identify provider type	HCPCS codes: 99201–99205, 99212–99215, 99241–99245, 99251–99255, 99354–99357, 99401–99404, 99406-99407
Endoscopies	ICD-9 procedures: 45.11–45.14, 45.16, 45.21–45.27, 48.23, 48.24; HCPCS codes 43234, 43235, 43239, 44360, 44361, 44376, 44378, 45335, 45378, 45379, 45380, 45381, 45382, 45383, 45384, 45385, 45300, 45303, 45305, 45307, 45308, 45309, 45315, 45317, 45320, 45321, 45327, 45330–45335, 45337–45340, 45345
IBD surgeries	ICD-9 procedure codes: 45.7, 45.71, 45.73–45.76, 45.79, 45.8, 45.9, 45.90–45.95, 45.6, 45.61–45.63, 46.0, 46.01–46.04,46.1, 46.10–46.11, 46.13–46.14, 46.2, 46.20–46.24, 48.5, 48.6, 48.61–48.65, 48.69

## References

[1] van Assche G, Vermeire S, Rutgeerts P (2010). Mucosal healing and anti TNFs in IBD. Curr Drug Targets.

[2] Lichtenstein G, Hanauer S, Sandborn W (2009). Practice Parameters Committee of the American College of Gastroenterology. Management of Crohn's Disease in Adults. Am J Gastroenterol.

[3] Kornbluth A, Sachar D (2010). Practice Parameters Committee of the American College of Gastroenterology. Ulcerative colitis practice guidelines in adults: American College Of Gastroenterology, Practice Parameters Committee. Am J Gastroenterol.

[4] Curkovic I, Egbring M, Kullak-Ublick G (2013). Risks of inflammatory bowel disease treatment with glucocorticosteroids and aminosalicylates. Dig Dis.

[5] Allen J, Dassopoulos, T, Brill, JV, et al. Adult Inflammatory Bowel Disease Physician Performance Measures Set. In: Institute AGAA, ed: American Gastroenterological Association 2011:1–52.

[6] Sarnes E, Crofford L, Watson M, Dennis G, Kan H, Bass D (2011). Incidence and US costs of corticosteroid-associated adverse events: a systematic literature review. Clin Ther.

[7] Bewtra M, Johnson F (2013). Assessing patient preferences for treatment options and process of care in inflammatory bowel diseas: a critical review of quantitative data. Patient.

[8] Shah M, Chaudhari S, Mclaughlin TP, Kan HJ, Bechtel B, Dennis GJ (2013). Cumulative burden of oral corticosteroid adverse effects and the economic implications of corticosteroid use in patients with systemic lupus erythematosus. Clin Ther.

[9] Sherman M, Tsynman DN, Kim A, Arora J, Pietras T, Messing S, et al. Sustained improvement in health-related quality of life measures in patients with inflammatory bowel disease receiving prolonged anti-tumor necrosis factor therapy. J Dig Dis. 2013. 10.1111/1751-2980.12125 24373601

[10] Rogler G, Vavricka S, Schoepfer A, Lakatos PL (2013). Mucosal healing and deep remission: what does it mean?. World J Gastroenterol.

[11] Lichtenstein G, Yan S, Bala M, Hanauer S (2003). Remission in patients with crohn's disease is associated with improvement in employment and quality of life and a decrease in hospitalizations and surgeries. Am J Gastroenterol.

[12] Allen P, Peyrin-Biroulet L (2013). Moving towards disease modification in inflammatory bowel disease therapy. Curr Opin Gastroenterol.

[13] Feagan BG, Lemann M, Befrits R, Connell W, D'Haens G, Ghosh S (2012). Recommendations for the treatment of Crohn's Disease with tumor necrosis factor antagonists: an expert consensus report. Inflamm Bowel Dis..

[14] Park K, Bass D (2011). Inflammatory bowel disease-attributable costs and cost-effective strategies in the United States: a review. Inflamm Bowel Dis.

[15] Lichtenstein GR, Abreu M, Cohen R, Tremain W (2006). American Gastroenterological Association Institite medical position statement on corticosteroids, immunomodulators, and infliximab in inflammatory bowel disease. Gastroenterology.

[16] American Gastroenterological Association. Adult Inflammatory Bowel Disease Physician Performance Measures Set. 2011; http://www.gastro.org/practice/quality-initiatives/IBD_Measures.pdf. Accessed April 4, 2013.

[17] Juneja M, Baidoo L, Schwartz MB, Barrie A, Regueiro M, Dunn M (2012). Geriatric inflammatory bowel disease: phenotypic presentation, treatment patterns, nutritional status, outcomes, and comorbidity. Dig Dis Sci.

[18] Kannel W (2000). Incidence and epidemiology of heart failure. Heart Fail Rev.

[19] Berger N, Savvides P, Korukian SM (2006). Cancer in the elderly. Trans Am Clin Climatol Assoc.

[20] Rommer PS, Buckow K, Ellenberger D, Friede T, Pitschnau-Michel D, Fuge J (2015). Patients characteristics influencing the longitudinal utilization of steroids in multiple sclerosis--an observational study. Eur J Clin Invest.

[21] Foote SB, Halaas GW (2006). Defining a Future For Fee-For-Service Medicare. Health Aff.

[22] Herrinton LI, Liu L, Weng X, Lewis JD, Huftless S, Allison JE (2011). Role of thiopurine and anti-TNF therapy in lymphoma in inflammatory bowel disease. Am J Gastroenterol.

[23] Liu L, Allison J, Herrinton L (2009). Validity of computerized diagnoses, procedures, and drugs for inflammatory bowel disease in northern California managed care organization. Pharmacoepidemiol Drug Saf.

[24] Joynt K, Orav E, Jha A (2011). The association between hospital volume and processes, outcomes, and costs of care for congestive heart failure. Ann Intern Med.

[25] Healthcare Cost and Utilization Project, Comorbidity Software, version 3.3. 2008.

[26] Vong S, Bell B (2004). Chronic liver disease mortality in the United States, 1990–1998. Hepatology.

[27] MEDPac. Report to the Congress: Medicare and the Health Care Delivery System. 2012.

[28] Bryson CL, Au DH, Young B, McDonell MB, Fihn SD (2007). A refill adherence algorithm for multiple short intervals to estimate refill compliance (ReComp). Med Care.

[29] Centers for Medicare and Medicaid Services (2012). Medicare Claims Processing Manual, Chapter 17: Drugs and Biologicals.

[30] USDA Economic Research Service. Rural Urban Commuting Area Codes. 2005; http://www.ers.usda.gov/data-products/rural-urban-commuting-area-codes.aspx. Accessed April 4, 2013.

[31] American Gastroenterological Association Insititute. IBD CPT Codes Ulcerative Colitis & Crohn's Disease. In: Association TAG, ed. http://www.gastro.org/practice/clinical-service-line/525-325PNQ_13-2_PDF_Document_Updates_CPT_Codes-_IBD_Final.pdf, Accessed April 11, 2014.

[32] Cairns SR, Scholefield JH, Steele RJ, Dunlop MG, Thomas HJW, Evans GD (2010). Guidelines for colorectal cancer screening and surveillance in moderate and high risk groups (update from 2002). Gut..

[33] Wang YR, Cangemi JR, Loftus EV, Picco MF (2013). Rate of early/missed colorectal cancers after colonoscopy in older patients with or without inflammatory bowel disease in the united states. Am J Gastroenterol.

[34] Bewtra M, Su C, Lewis J (2007). Trends in hospitalization rates for inflammatory bowel disease in the United States. Clin Gastroenterol Hepatol.

[35] Charlson ME, Pompei P, Ales KL, Mackenzie CR (1987). A new method of classifying prognostic comorbidity in longitudinal studies: development and validation. J Chronic Dis.

[36] Hilbe J (2011). Negative Binomial Regression.

[37] Senturk D, Dalrymple LS, Mu Y, Nguyen DV (2014). Weighted hurdle regression method for joint modeling of cardiovascular events likelihood and rate in the US dialysis population. Stat Med.

[38] Rose CE, Martin SW, Wannemuehler KA, Plikaytis BD (2006). On the use of zero-inflated and hurdle models for modeling vaccine adverse event count data. J Biopharm Stat.

[39] Rogers E (2003). Diffusion of Innovations.

[40] Grootendorst P (1995). A comparison of alternative models of prescription drug utilization. Health Econ.

[41] Al Hadithy AFY (2005). deBoer NKH, Derijks LJJ, Eschar JC, Mulder CJJ, Brouwers JRBJ. Thiopurines in inflammatory bowel disease: pharmacogenetics, therapeutic drug monitoring and clinical recommendations. Dig Liver Dis.

[42] Chevaux J, Peyrin-Biroulet L, Sparrow M (2011). Optimizing thiopurine therapy in inflammatory bowel disease. Inflamm Bowel Dis.

